# Optimization of Cholinesterase-Based Catalytic Bioscavengers Against Organophosphorus Agents

**DOI:** 10.3389/fphar.2018.00211

**Published:** 2018-03-13

**Authors:** Sofya V. Lushchekina, Lawrence M. Schopfer, Bella L. Grigorenko, Alexander V. Nemukhin, Sergei D. Varfolomeev, Oksana Lockridge, Patrick Masson

**Affiliations:** ^1^Laboratory of Computer Modeling of Bimolecular Systems and Nanomaterials, N. M. Emanuel Institute of Biochemical Physics of Russian Academy of Sciences, Moscow, Russia; ^2^Department of Biochemistry and Molecular Biology, Eppley Institute, University of Nebraska Medical Center, Omaha, NE, United States; ^3^Chemistry Department, Lomonosov State University, Moscow, Russia; ^4^Neuropharmacology Laboratory, Kazan Federal University, Kazan, Russia

**Keywords:** bioscavenger, organophosphorus compound, computer design, phosphotriesterase, acetylcholinesterase, butyrylcholinesterase

## Abstract

Organophosphorus agents (OPs) are irreversible inhibitors of acetylcholinesterase (AChE). OP poisoning causes major cholinergic syndrome. Current medical counter-measures mitigate the acute effects but have limited action against OP-induced brain damage. Bioscavengers are appealing alternative therapeutic approach because they neutralize OPs in bloodstream before they reach physiological targets. First generation bioscavengers are stoichiometric bioscavengers. However, stoichiometric neutralization requires administration of huge doses of enzyme. Second generation bioscavengers are catalytic bioscavengers capable of detoxifying OPs with a turnover. High bimolecular rate constants (*k*_cat_/*K*_*m*_ > 10^6^ M^−1^min^−1^) are required, so that low enzyme doses can be administered. Cholinesterases (ChE) are attractive candidates because OPs are hemi-substrates. Moderate OP hydrolase (OPase) activity has been observed for certain natural ChEs and for G117H-based human BChE mutants made by site-directed mutagenesis. However, before mutated ChEs can become operational catalytic bioscavengers their dephosphylation rate constant must be increased by several orders of magnitude. New strategies for converting ChEs into fast OPase are based either on combinational approaches or on computer redesign of enzyme. The keystone for rational conversion of ChEs into OPases is to understand the reaction mechanisms with OPs. In the present work we propose that efficient OP hydrolysis can be achieved by re-designing the configuration of enzyme active center residues and by creating specific routes for attack of water molecules and proton transfer. Four directions for nucleophilic attack of water on phosphorus atom were defined. Changes must lead to a novel enzyme, wherein OP hydrolysis wins over competing aging reactions. Kinetic, crystallographic, and computational data have been accumulated that describe mechanisms of reactions involving ChEs. From these studies, it appears that introducing new groups that create a stable H-bonded network susceptible to activate and orient water molecule, stabilize transition states (TS), and intermediates may determine whether dephosphylation is favored over aging. Mutations on key residues (L286, F329, F398) were considered. QM/MM calculations suggest that mutation L286H combined to other mutations favors water attack from apical position. However, the aging reaction is competing. Axial direction of water attack is not favorable to aging. QM/MM calculation shows that F329H+F398H-based multiple mutants display favorable energy barrier for fast reactivation without aging.

## Introduction

Organophosphorus agents (OPs) are irreversible inhibitors of acetylcholinesterase (AChE) and are dreadful poisons. They occur as pesticides (such as parathion/paraoxon, dichlorvos, chlorpyrifos, malathion, etc.), chemical warfare nerve agents (CWNAs such as tabun, sarin soman, and V-agents), active metabolites of synthetic engine oil components (such as cresylsaligenyl phosphate, the metabolite of tri-o-cresylphosphate), and certain drugs (echothiophate, metriphonate, etc.). OPs are among the most dreadful poisons. Though, chemical warfare agents (CWA) have been banned, they still represent a threat, and have recently been used in terrorist attacks and assassinations (Worek et al., 2016b; Patocka, 2017). Accidental and intentional self-poisoning by OP pesticides cause some 3,000,000 intoxications and more than 200,000 deaths per year, worldwide. This makes these agents major public health concerns, especially in developing countries (Eddleston et al., 2008).

Acute OP poisoning causes a major cholinergic syndrome, leading to acute respiratory failure, seizures, and cardio-vascular distress (Eddleston et al., 2008). The current medical counter-measures for protection against acute OP poisoning combine pre-treatment and post-exposure strategies. Pre-treatment involves administration of carbamates (e.g., pyridostigmine, physostigmine, etc.) that transiently inhibit AChE (Myhrer and Aas, 2016). However, these drugs do not confer a full protection (prophylaxis). Pyridostigmine does not cross the blood brain barrier, and therefore only protects peripheral AChE. Physostigmine and scopolamine do cross the blood brain barrier, and thus, protect central AChE. However, carbamates possibly cause cognitive, neurobehavioral side effects and perturb thermoregulation. These side-effects have to be corrected by the use of adjunct drugs (Myhrer and Aas, 2016). Post-exposure treatment for acute poisoning is based on the implementation of AChE nucleophilic reactivators (oximes such as 2-PAM, obidoxime, HI-6), antimuscarinic drugs (atropine), and anticonvulsants (diazepam, midazolam acting as neuroprotectants). These drugs mitigate the acute effects of lethal doses but have limited action against OP-induced irreversible brain damage. Other drugs (anti-nicotinic drugs, neuroprotectants, antioxidants, etc.) are under consideration for delayed treatment and prevention of neurological sequellae (Masson, 2016b). However, in the past 30 years no major improvement has been made in classical pharmacological treatment of OP poisoning. Some progress has resulted from better use of existing drugs, better management of chemical casualties (Myhrer and Aas, 2016; Rice et al., 2016), and encapsulation of quaternary oximes in nanoparticles (Pashirova et al., 2017). However, new potential drugs such as anti-Alzheimer inhibitors of AChE, non-quaternary oximes, anti-nicotinic drugs, and neuroprotectants like ketamine are still under evaluation (Masson, 2016b).

## Bioscavenger concept

Bioscavengers are the most appealing alternative/complementary therapeutic approach. Initially, they were considered for prophylaxis of NA poisoning (Doctor and Saxena, 2005; Lenz et al., 2005). Subsequently, their use in post-exposure treatment has proven to be effective (Mumford et al., 2013). These biopharmaceuticals can be administered by injection or inhalation, or they can be produced *in vivo* through gene delivery vectors. Bioscavengers are biopharmaceuticals capable of sequestering and inactivating highly toxic compounds. Bioscavengers against OPs can be enzymes, antibodies, or reactive proteins. There are of three types: (a) stoichiometric bioscavengers that react mole-to-mole with OPs; (b) pseudocatalytic bioscavengers that are a combination of stoichiometric bioscavengers acting together with a reactivator; (c) catalytic bioscavengers that use OPs as substrates, degrading these toxic molecules via a turnover process that releases non-toxic products. Several endogenous stoichiometric [butyrylcholinesterase (BChE), carboxylesterases] and catalytic bioscavengers [paraoxonase-1 (PON-1)] are present in the skin, blood, and liver. These participate in natural defense against OPs, by reducing the transfer of OP molecules to physiological targets and depot sites (for a review on endogenous bioscavengers, see Masson, 2016a). Exogenous bioscavengers neutralize, i.e., detoxify, OP molecules in the blood stream before they reach physiological targets (for recent reviews on bioscavengers see Nachon et al., 2013; Masson, 2016b).

The first requirement for an operational bioscavenger is that the neutralization reaction must be fast to prevent reaction with physiologically sensitive targets. Even in the most serious cases of poisoning, the concentration of OPs in blood is low. As a consequence, the OP concentration is much lower than the dissociation constant (*K*_*d*_) of complexes between OP and stoichiometric bioscavengers, or the Michaelis constant (*K*_*m*_) of catalytic bioscavengers. Thus, the rate of detoxification (d[P]/dt) occurs under first-order conditions. The reaction kinetics are, therefore, controlled by the bimolecular rate constant (*k*_II_) and the concentration of bioscavenger [E] in blood (Equation 1).

(1) $$\begin{array}{r} \text{d}\left[\text{P}\right]/\text{dt}={\text{k}}_{\text{II}}\cdot \left[\text{E}\right]\cdot \left[\text{OP}\right] \end{array}$$

In Equation (1), *k*_II_ = *k*_p_/ *K*_*d*_ for a stoichiometric bioscavenger, *k*_II_ = *k*_cat_/*K*_*m*_ for a catalytic bioscavenger. This makes the product, *k*_II_·[E], a first order rate constant (Masson and Lushchekina, 2016).

Other requirements for a successful bioscavenger are the following: (1) bioscavengers must display a slow blood clearance, (2) they must not induce immune response, (3) they must not induce secondary effects, and (4) they must be easily and feasibly deliverable. The last three requirements can be addressed by encapsulation into nanocontainers, or by chemical modification (capping). In addition, bioscavengers must be prepared under GMP conditions. They must not contain infectious organisms, infectious viruses, endotoxins, or protein contaminants. Lastly, they must be stable upon storage. Bioscavengers studied to date have a narrow specificity/enantioselectivity. Therefore, a mixture of several bioscavengers will need to be used to cover a wide spectrum of NAs (Masson, 2016a; Worek et al., 2016a).

### Stoichiometric bioscavengers

First generation bioscavengers are stoichiometric bioscavengers. Among them, human BChE has proven to be safe and effective when challenged with multiple LD_50_ of NAs (Lockridge, 2015). However, stoichiometric neutralization of NAs needs huge doses e.g., 200 mg of BChE for protection of a person (70 kg) against 2 × LD_50_ of soman. Production of such large quantities of BChE is extremely expensive. Though, a new affinity chromatography method for purification of BChE (Onder et al., 2017) and recent functional bacterial expression of human AChE (Goldenzweig et al., 2016) and BChE (Brazzolotto et al., 2017) are expected to significantly reduce the cost of production, the use of human BChE, even for protection of first responders, will remain extremely expensive.

### Pseudocatalytic bioscavengers

A variation on the stoichiometric theme involves co-administration of special stoichiometric bioscavengers—e.g., cholinesterase mutants not susceptible to “aging” after phosphylation—with fast-reactivating oximes. This produces pseudo-catalytic bioscavengers (Radić et al., 2013; Kovarik et al., 2015). However, pharmacokinetic compatibility between enzyme and reactivator is an issue.

### Catalytic bioscavengers

Second generation bioscavengers are catalytic bioscavengers capable of detoxifying NAs with a high turnover. If a catalytic bioscavenger has a high bimolecular rate constant (*k*_cat_/*K*_*m*_ > 10^6^ M^−1^min^−1^), then much lower doses, compared to stoichiometric bioscavengers, would be needed for rapid detoxification of NAs. For example, a dose of 10 mg of a 40 kDa catalytic bioscavenger would give [E] = 1 μM in plasma. If *k*_cat_/*K*_*m*_ > 5 × 10^7^ M^−1^·min^−1^ then the half time for complete hydrolysis of OP would be <1 s (Worek et al., 2016a). To date, the most promising catalytic bioscavengers are evolved enantioselective phosphotriesterases (PTEs), such as evolved *Brevundimonas* (*Pseudomonas) diminuta* PTE and evolved paraoxonase-1 (PON-1) (Ashani et al., 2016). The evolved mutant of *B. diminuta* PTE, L7ep-3, displays *k*_cat_/*K*_*m*_ = 4.8 × 10^7^ M^−1^·min^−1^ for S_p_ VX and *k*_cat_/*K*_*m*_ = 1.6 × 10^5^ M^−1^·min^−1^ for S_p_ VR (Bigley et al., 2015). Other mutants of this enzyme, made by directed evolution and computer design, display a wide spectrum of activity. Not only do they hydrolyze V agents with high efficiency, but also G agents with *k*_cat_/*K*_*m*_ > 5 × 10^7^ M^−1^·min^−1^ for tabun and sarin (Goldsmith et al., 2017). The evolved mutant of PON-1, C23AL, has *k*_cat_/*K*_*m*_ = 1.2 × 10^7^ M^−1^·min^−1^ for racemic VX (Goldsmith et al., 2016). A dose of 2 mg/kg PON-1 was found to protect against 2 × LD_50_ of VX in guinea pig (Wille et al., 2016).

In light of these recent successes, research on novel catalytic bioscavengers is continuing. Different approaches have been implemented: (a) Screening of extreme biotopes has already led to highly stable promiscuous PTE/PLLs that are appealing for decontamination of skin and sensitive materials (Jacquet et al., 2016; Restaino et al., 2017). (b) Mining genomic and structural databases has identified sequences/structures that can potentially hydrolyze OPs (Hiblot et al., 2015; Jacob et al., 2016). (c) Research into OP degradation by known OP-reactive enzymes is aimed at improving their catalytic properties in order to convert them into efficient OP hydrolases (OPases). Different types of enzymes have been investigated: prolidases, oxidases (laccases, glutathione transferases, etc.) carboxylesterases, and cholinesterases (ChEs). For reviews on these approaches see Nachon et al. (2013) and Masson and Nachon (2017).

## ChE-based catalytic bioscavengers

**Scheme 1:**
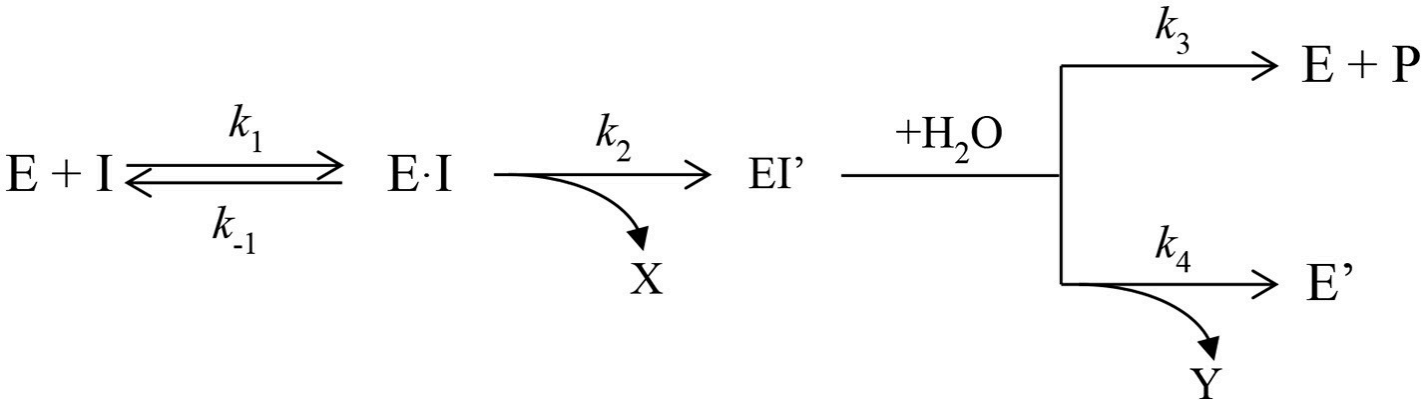
Inhibition of ChEs by Ops.

Because OPs are hemi-substrates for ChEs (Scheme 1), ChEs are attractive starting points for conversion into OPases. After rapid formation of a reversible enzyme·OP complex (*k*_1_ is the association constant for formation of non-covalent complex between enzyme and OP, and k_−1_ the dissociation of the reversible michaelian complex), phosphylation (*k*_2_) of ChEs' active site serine is fast. However, in most cases, water-mediated dephosphylation (*k*_3_) is very slow or impossible. In addition, there is a second post-inhibitory reaction, i.e., dealkylation (*k*_4_), of the bound adduct which releases an alkoxy chain (Y). This reaction is referred to as “aging.” “Aging” makes nucleophilic attack on the phosphorus atom by water or even by strong nucleophilic compounds impossible (for a review on aging see Masson et al., 2010). To make a ChE-based catalytic bioscavenger, the dephosphylation rate constant (*k*_3_) has to be increased by several orders of magnitude.

### Early studies

**Figure 1:**
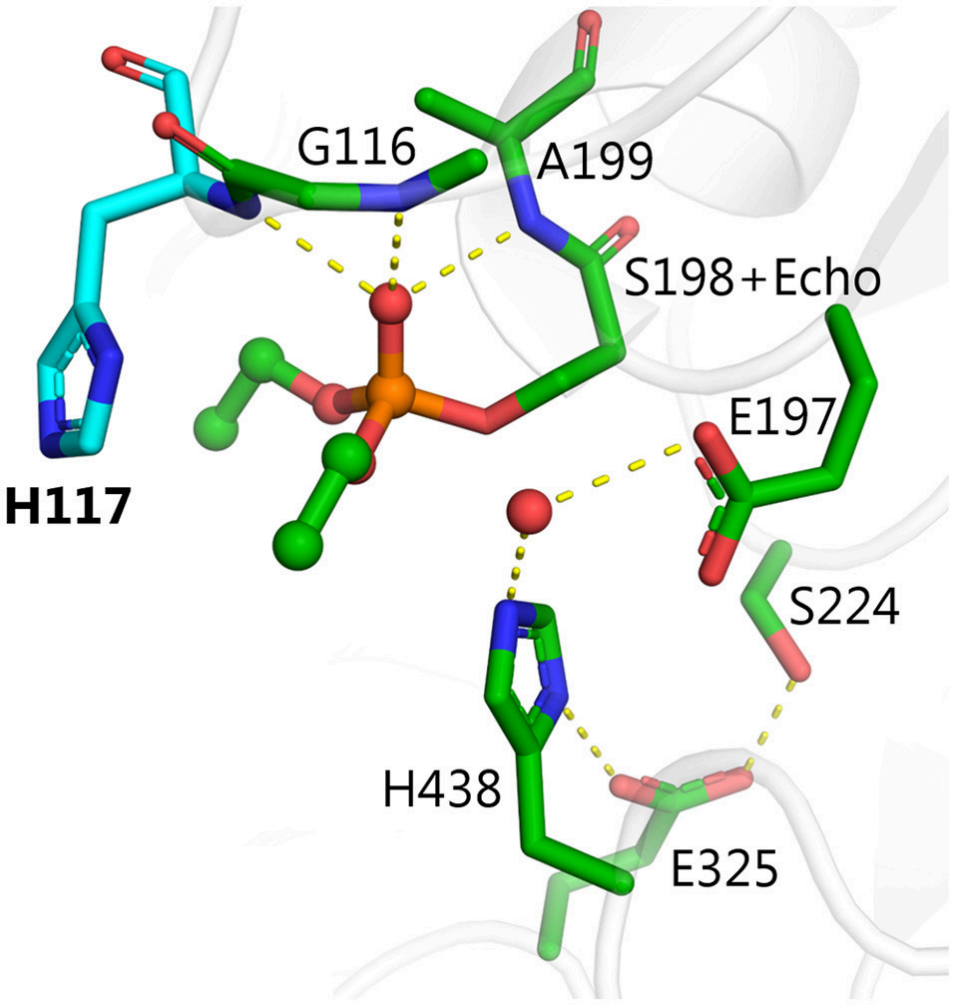
X-ray structure of phosphorylated G117H active site [PDB ID 2XMD (Nachon et al., 2011)]. Carbon atoms of the mutated residue, H117, are shown in cyan, atoms of echothiophate residue conjugated to catalytic serine 198, and crystallographic water closest to the phosphorus atom are shown as balls. Yellow dashes show critical hydrogen bonds in the active site.

Attempts to convert ChEs into effective OPases have been underway for more than two decades. Dephosphylation of the ChE-OP adduct is slow because the active site is constructed to promote attack by water from the side of the Ser_198_-O–P adduct (roughly 90° relative to the P–O bond), whereas release of the phosphate requires attack on the phosphorus from the side opposite to the serine (roughly 180° relative to the P–O bond) (Jarv, 1984). The first attempts to improve dephosphorylation of OP-inhibited BChE involved introduction of a nucleophilic group into the oxyanion hole so that nucleophilic attack on the phosphorus atom could occur from the side of the phosphorous opposite to S198. Mutants were designed from the modeled 3D structure of human BChE. The first successful mutant replaced glycine at position 117 with histidine, G117H (Millard et al., 1995). Resolution of the crystal structure of human BChE (Nicolet et al., 2003), and later of the mutant G117H (Figure 1) confirmed the validity of the proposed structure (Nachon et al., 2011).

The G117H mutant behaves like an OPase with *k*_3_ = 1.3 min^−1^ with paraoxon as substrate, 1.2 min^−1^ with echothiophate (Lockridge et al., 1997) 0.0052 min^−1^ with sarin, and 0.0072 min^−1^ with VX (Millard et al., 1995). Other mutations were subsequently introduced to slow down the aging process (Millard et al., 1998; Broomfield et al., 1999). However, attempts to speed up *k*_3_ and to improve *k*_cat_ by introducing other mutations, failed. More than 60 mutants of human BChE and AChE were made and compared to other self-reactivating ChEs and carboxylesterase, using echothiophate as inhibitor (Table 1; published and unpublished data). Several of these other mutants could be classified as OPases, but none were more active than G117H. Moreover, there was no conclusive answer for the mechanism of reactivation by these mutants. For a review on these early studies, see Masson et al. (2008).

**Table 1:** Reactivation parameters for cholinesterase mutants inhibited by echothiophate.

**#**	**Enzyme**	**Concept to be tested**	***k*^a^_dephos_ min^−1^**	**Reactivation %**	**Aging *t*_1/2_**	**Activity^b^ unit/ml**
1	WT	–	<0.0003	3	–	4.4
2	G117H^c^	–	1.15–1.3	100	5.5 h	1.1
3	G117H/G115H	Additional positive charge	0	–	–	0.011
4	G117H/G439H	“	0	–	–	0.006
5	G117H/V288H	“	??	–	–	0.060
6	G117H/A199H	“	0	–	–	0.007
7	G117H/L286H	“	<0.0072	??	–	0.009
8	G117H/A328H	“	Yes	–	–	0.010
9	G117H/F329H	“	Yes	–	–	0.010
10	V288H/F329D	Ion-pairing	Yes	–	–	0.030
11	V288H/F329E	“	??	–	–	0.015
12	V288H/G291D	“	0	–	–	0.13
13	G117H/F329E/V288A	“	Yes	–	–	0.012
14	L286H^d^	Alternate His position^g^	0.0032	79	–	0.36
15	L286GHG^d^	“	0.0016	50	–	0.18
16	L286HG^d^	“	0.0010	44	–	0.017
17	L286GH^d^	“	0.0024	96	–	0.030
18	V288H	“	0	–	–	0.71
19	V288GH	“	0	–	–	0.040
20	V288GHG	“	0	–	–	0.058
21	Q119H	“	0	–	–	0.34
22	T120H	“	0	–	–	0.025
23	A277H	“	0	–	–	4.9
24	G117C	Mechanism premise	??	–	–	0.14
25	G117K	“	0	–	–	2.6
26	G117S	“	0	–	–	2.3
27	G117E^d^	“	0.0037	50	–	0.052
28	G117Y	“	0	–	–	0.032
29	G117L	“	0	–		0.006
30	G117H/Q119E	H-bond acceptor	<0.0016	8	–	0.48
31	G117H/A199E^e^	“	0	–	–	0.11^e^
32	G117H/L286Y	“	0	–	–	0.006
33	G117H/F329Y	“	Yes	–	–	0.008
34	F329E	“	0	–	–	0.32
35	F329S	“	0	–	–	2.1
36	K339M	“	0	–	–	4.0
37	F329S/E197Q	“	0	–	–	2.3
38	G117D	Blowfly	0.025	95	44 h	0.31
39	W231A	“	0.0009	63	–	0.13
40	F398I	Cat	0	–	–	0.063
41	G117H/F398I	“	??	–	–	0.021
42	P285L	“	0	–	–	0.13
43	P285L/F398I	“	0	–	–	0.060
44	G117H/P285L	“	??	–	–	0.018
45	G117H/P285L/F398I	“	<0.0155	10	–	0.021
46	G225A	*Drosophila*	<0.0001	11	–	0.035
47	L286Y	“	0	–	–	0.017
48	G225A/L286Y	“	0	–	–	0.009
49	G117H/E197Q^d^	Aging modifications	0.024	89	15.5 h	3.3
50	G117H/E197H	“	0.0003	50	–	0.008
51	G117H/E197D^d^	“	0.084	100	17 h	2.6
52	G117H/E197G^d^	“	0.014	100	>50 h	2.8
53	G117H/W82F^d^	“	0.17	85	13 h	0.74
54	E197Q	“	0	–	–	3.2
55	G117H/W82A^f^	“	??	–	–	5.6^d^
56	G117H/Y332A	Miscellaneous	??	–	–	0.020
57	W430A	“	0	–	–	1.3
58	F329S/E197Q	“				
59	E325Q	Active site	0	–	–	–
60	E325Q/G117H	“	0	–	–	–
61	G117H/A328W	“	?			
62	G117H/A328G	(-)Cocaine hydrolysis	0.60	76	33 h	
63	G117H/L286R	Rat	Yes	–	–	–
64	G122H/Q124Y/S125T^h^	*Bungarus fasciatus*	Yes^h^	–	–	–

a *k_dephos_ = k_reactivation_ – k_aging_ [for theoretical bases and experimental details see Schopfer et al. (2004) and Dafferner et al. (2017)]. When the word “yes” appears instead of a rate, the existence of dephosphorylation was clearly indicated by the assay for progressive inhibition in the presence of substrate, but there was not sufficient substrate activity to permit the measurement of spontaneous reactivation and determination of the rate of dephosphorylation. When “??” appears instead of a rate, the progressive inhibition assay results were equivocal*.

b *These values indicate the rate of butyrylthiocholine hydrolysis in the stock solution. Standard assay conditions consisted of 1 mM butyrylthiocholine, 0.5 mM DTNB in 0.1 M potassium phosphate buffer, pH 7.0, at 25°C. The activities are presented here to give an indication of the levels of activity from which determinations of echothiophate hydrolysis were made. Since the concentration of the BChE mutant in these media was not determined, the relative activity values do not represent the relative turnover numbers*.

c *Lockridge et al. (1997)*.

d *Schopfer et al. (2004)*.

e *Assayed with o-nitrophenylbutyrate and inhibited by paraoxon. There was no measurable activity with 1 mM butyrylthiocholine and no inhibition with 2 mM echothiophate in the presence of 1 mM butyrylthiocholine*.

f *Assayed with o-nitrophenylbutyrate and echothiophate. There was no measurable activity with butyrylthiocholine*.

g *The combinational approach generated self-reactivating human BChE mutants bearing His in similar positions, i.e., 284, 285, 286, and 287 Terekhov et al. (2017)*.

h *k_cat_/K_m_-values for human G117H BChE are 7, 90, and 420 times faster than k_cat_/K_m_-values for the Bungarus fasciatus G122H/Y124Q/S125T mutant with DFP, paraoxon, and echothiophate, respectively Poyot et al. (2006)*.

The lesson from this early work is that knowledge of the ChE 3D structure is necessary but not sufficient for making mutants displaying the desired catalytic properties. Increasing the dephosphylation rate constant implies lowering the energy barrier of dephosphylation. This can be achieved either by chance, i.e., by making numerous random mutations, or rationally from knowledge of the reaction energy profiles, the structure of transition states (TS), and the molecular dynamics of the mutants.

### New developments

Two new strategies for converting ChEs into fast OPases have been recently employed. One is based on a combinational approach and the other on computer re-design of ChEs. The combinational approach requires an *ad hoc* functional expression system and an adapted ultrahigh-throughput screening method. Expression of human BChE in *Pischia* and a microfluidic droplet technique using a fluorogenic substrate for BChE made it possible to screen a library of mutants (Terekhov et al., 2017). Several mutants that self-reactivate after inhibition by paraoxon were identified. These mutants bear multiple substitutions in the acyl-binding loop (between residues 284 and 288: which is TPLSV in wild-type BChE). Self-reactivation of these selected mutants is slow (*k*_3_ ~ 0.006 min^−1^). A histidine is present in the most efficient self-reactivatable mutants (HTIHG and PSHSG). These mutants will serve as starting materials for making improved mutants.

It is tempting to propose that the histidine in these mutants acts as a nucleophile capable of activating a water molecule. It is also conceivable that the mutations have altered the conformational flexibility of the acyl-binding loop, which in turn promotes reactivation. In that regard, it is known that pig BChE self-reactivates more rapidly than wild-type human BChE, after phosphonylation by VX (*k*_3_ = 0.025 min^−1^ with VX_S_) (Dorandeu et al., 2008). Based on molecular dynamic simulations this increased reactivation has been attributed to higher flexibility of the pig acyl-binding loop compared to that of human BChE (Brazzolotto et al., 2015). Recently, it was found that bovine BChE self-reactivates after phosphorylation by chlorpyrifos oxon (*k*_3_ = 0.023 min^−1^). Molecular dynamic simulations of “bovinated” human BChE in which 3 substitutions were made (G117S/P285L/F398I), showed that these three mutations are involved in self-reactivation by making the phosphorus atom more accessible to water. When mutation G117H was added to “bovinated” human BChE in the molecular dynamic simulations, accessibility of water to the phosphorus atom was further enhanced (Dafferner et al., 2017).

### Computational analysis

Self-reactivation of phosphorylated G117H BChE was studied in detail with molecular modeling methods (Albaret et al., 1998; Amitay and Shurki, 2009, 2011; Yao et al., 2012; Field and Wymore, 2014; Kulakova et al., 2015; Nemukhin et al., 2015). Several mechanisms were proposed. Both X-ray structure (Nachon et al., 2011) and QM/MM calculations (Kulakova et al., 2015; Nemukhin et al., 2015; Masson and Lushchekina, 2016) suggest that a water molecule is activated by E197 for nucleophilic attack on the phosphorus atom of the adduct, leading to formation of a pentacoordinate intermediate (PI). This is an associative mechanism. The role of H117 is to lower the energy barrier and stabilize the PI. Depending on the direction of proton transfer from H438, decomposition of the PI may, lead to either aged adduct or reactivated enzyme (cf. Scheme 1). Residue E197 is the key player in these processes. Double mutants, containing mutated E197 in addition to G117H have been shown to slow both the reactivation and aging processes (refer to mutants 49–52 in Table 1). Mutant G117H/E197Q is a potent member of this group. Because the E197Q mutation slows the aging process, soman-inhibited G117H/E197Q displays a higher fraction of reactivation relative to soman-inhibited G117H alone (Millard et al., 1998). Single mutations of Glu197 (E197D, E197Q, and E197G) all slowed down aging of di-isopropyl-phosphorylated BChE (Masson et al., 1997a). Moreover, X-ray structures of phosphonylated BChE show that activation of a water molecule by E197 determines the stereoselectivity of BChE for V-agents (Wandhammer et al., 2011).

**Figure 2:**
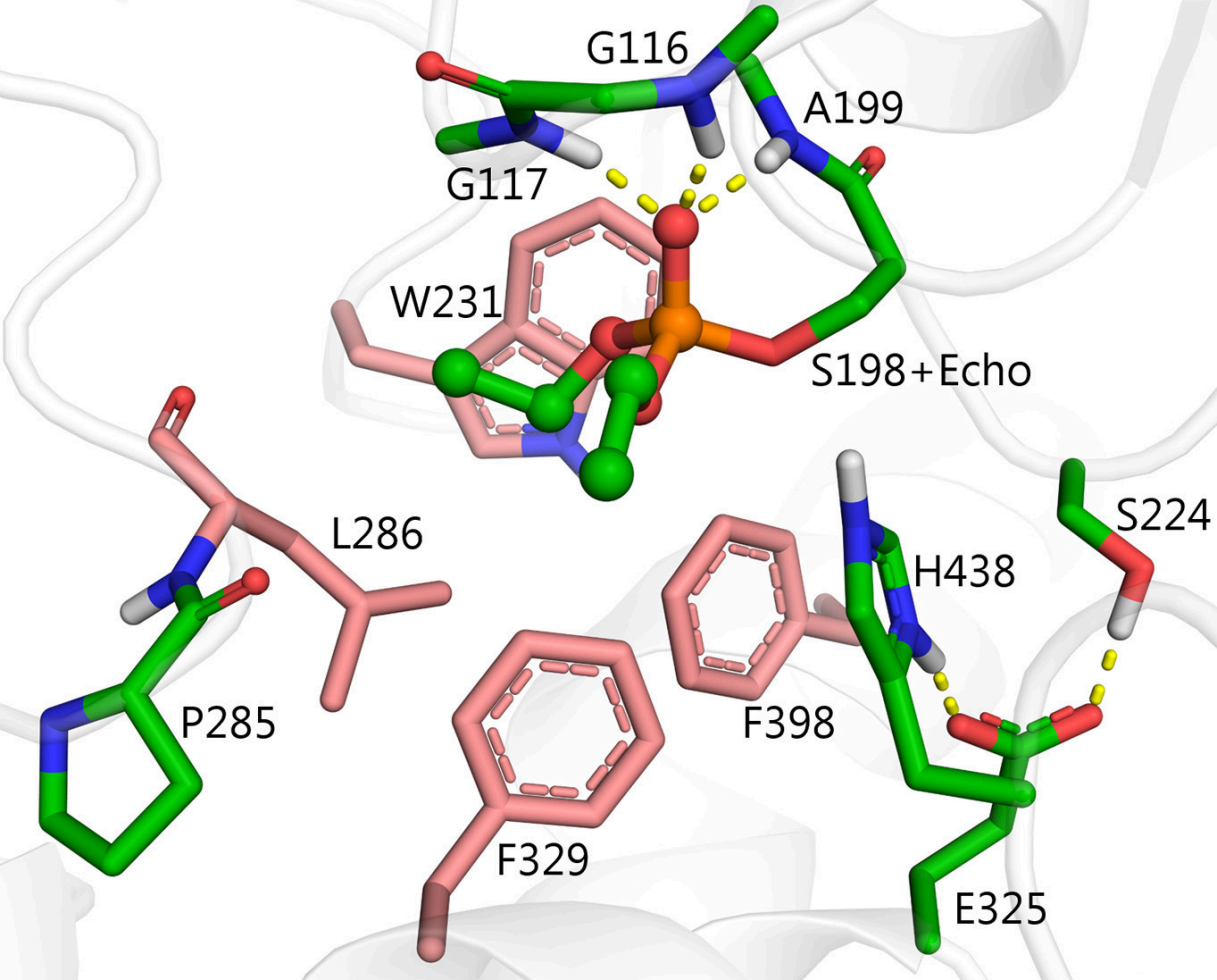
Structure of diethylphosphorylated wild-type BChE PDB ID 1XLW (Nachon et al., 2005), positions for introduction of alternative nucleophilic centers near the phosphorylated catalytic serine are shown in pink.

Among the mutants listed in Table 1, several were made in order to create hydrolysis mechanisms that do not involve G117H, i.e., L286H, V288H, Q119H, T120H, and A277H. Although L286H was the most effective of this group, none of these mutants was more effective than G117H. Inspection of the active site of BChE shows that residues L286, F329, F398, and W231 block the most effective, alternate positions for attack of a water molecule (Figure 2). Alternative nucleophilic centers were modeled into these areas. Modeling suggests that histidine should be used for such substitutions, rather than aspartic or glutamic acids. This is because the histidine p*K*_a_ value is higher than that of aspartic and glutamic acids, therefore histidine can be protonated more easily.

**Figure 3:**
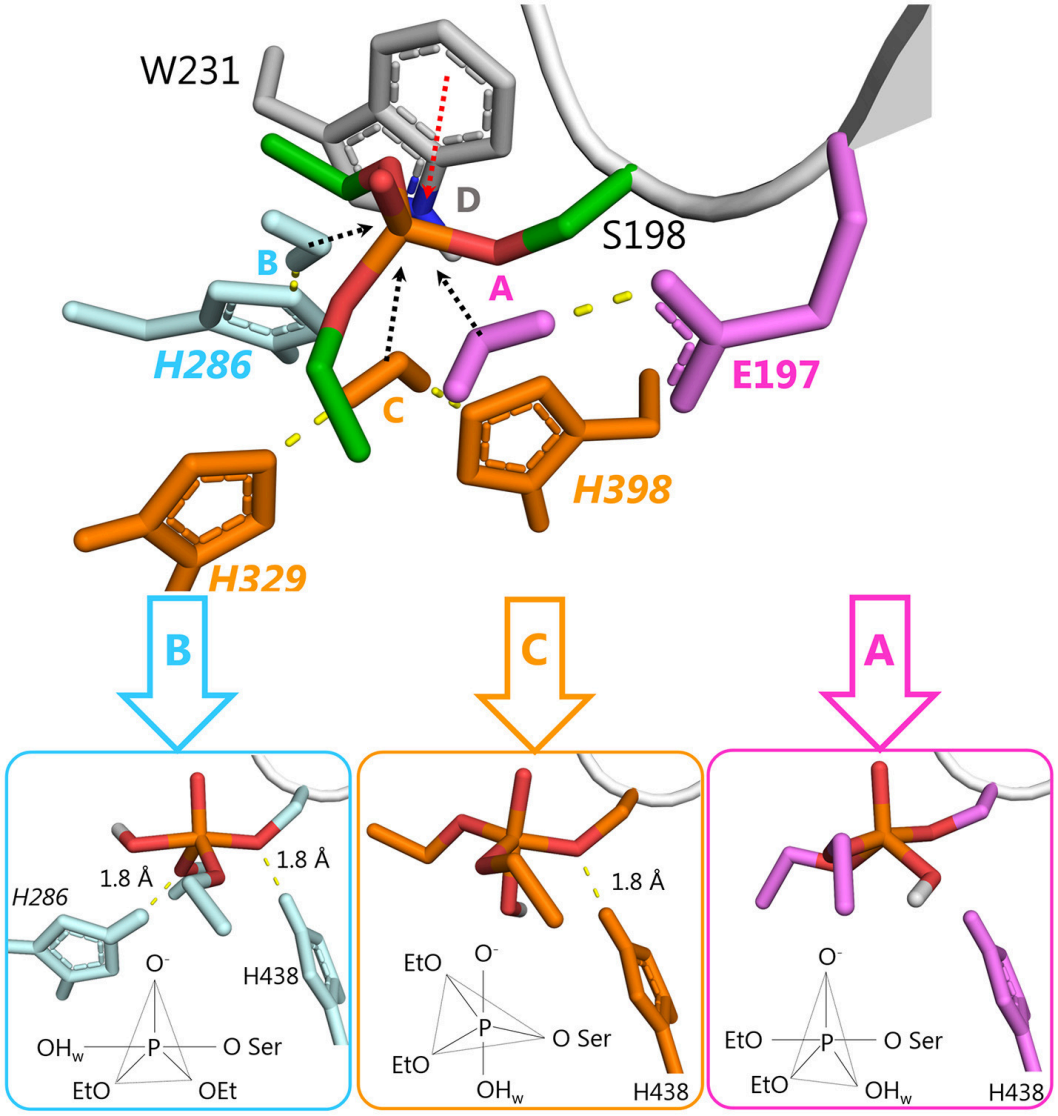
Possible directions for nucleophilic attack of a water molecule on the phosphorus atom of diethylphosphorylated serine with corresponding configurations of the resulting PI. 2D schemes indicate vertices of trigonal bipyramids. Atoms of the activating histidines and water molecules are colored according to the direction of the attack: direction A, magenta; direction B, cyan, direction C, orange.

There are four possible directions for nucleophilic attack of water molecules on the phosphorus atom (Figure 3). Direction A, adjacent to the serine residue, corresponds to the above-mentioned mechanism of aging and reactivation in wild-type and G117H BChE. Direction B from the apical position (opposite to the serine residue) could be realized with certain geometrical strain through introduction of a histidine into position 286. In the resulting PI, protonated H286 would form a hydrogen bond with an oxy-ethyl group from the diethylphosphate, suggesting strong competition between reactivation and aging processes. Direction C could be realized by introducing a histidine into position 398 and/or 329. Direction D is blocked by W231. Mutation W231A (mutation #39 in Table 1), leads to a low reactivation level, likely due to improved access of water molecules to phosphorus atom. Additional mutations like A202H or G225H that may improve efficiency of nucleophilic attack from this direction are not considered in this work.

**Figure 4:**
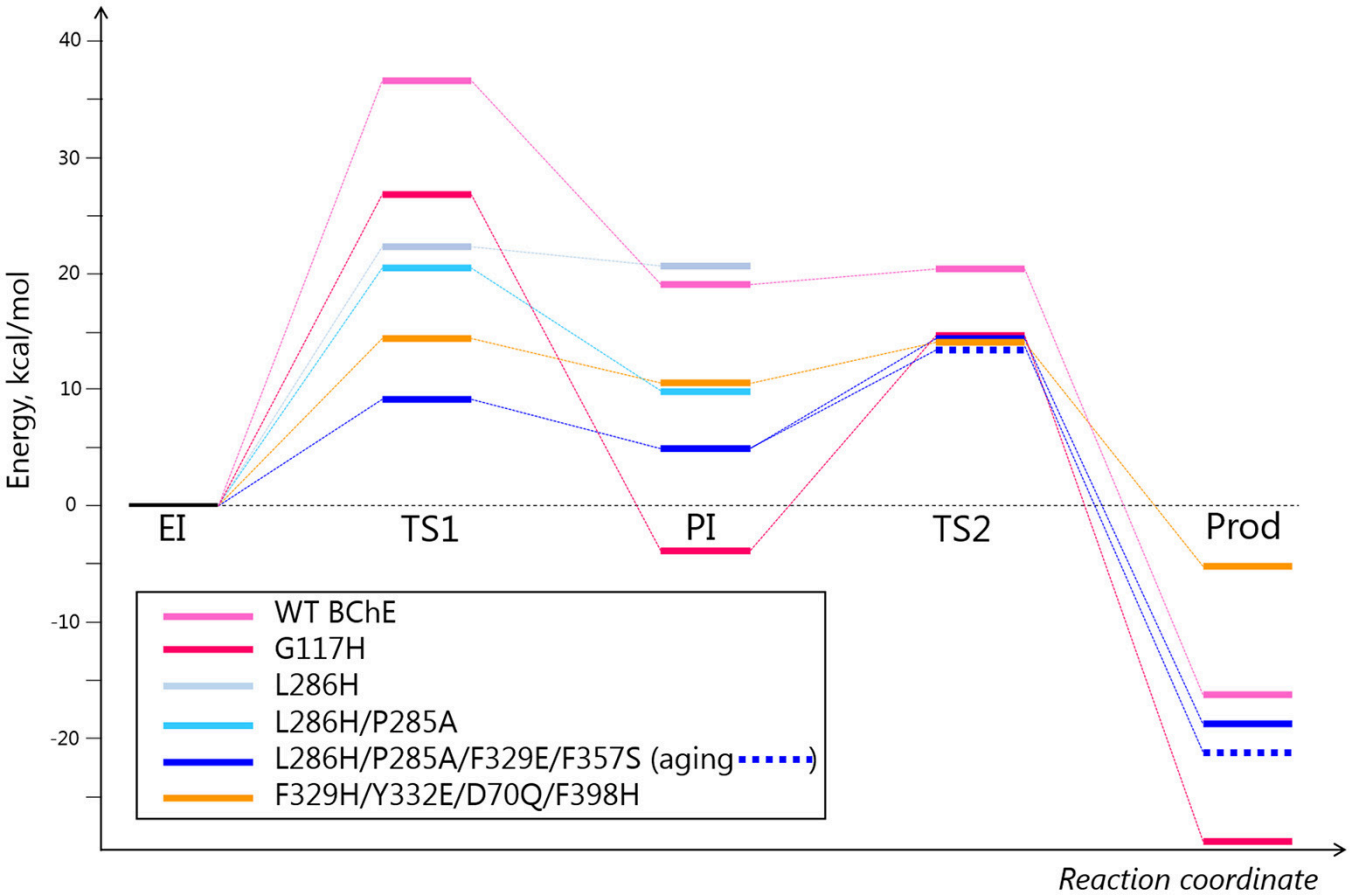
Energy diagrams for several selected mutants. Energy values are provided in Table 2. The pink, light blue, and orange colors correspond to different directions of nucleophilic attack depicted in Table 2 and Figure 3.

To analyze other possible pathways for hydrolysis of diethylphosphorylated BChE, QM/MM calculations were performed for two mutants from Table 1 (G117H and L286H) and for three newly proposed models (L286H/P285A, L286H/P285A/F329E/F357S, and F329H/Y332E/D70Q/F398H) (see Supplementary Information for methodological details and results are described below). Calculated energy levels of stationary points are listed in Table 2 and presented as a diagram in Figure 4.

**Table 2:** Calculated energy barriers and level of the pentacoordinated intermediate (PI) relative to the enzyme-inhibitor conjugate (EI) for selected mutants.

**Mutant**	**TS1**	**PI**	**TS2^c^**
WT BChE^d^	36.5	19.3	0.4
G117H^d^	26.7	−3.8	18.3
L286H	22.3	20.6	n.a.^e^
L286H/P285A	20.4	9.9	n.a.^e^
L286H/P285A/F329E/F357S	9.1	4.8	9.5 (8.3^f^)
F329H/Y332E/D70Q/F398H	15.9	10.8	3.5

*The values correspond to the diagram presented in Figure 4^a^^,^^b^*.

a *QM/MM calculations were performed with NwChem 6.5 software Valiev et al. (2010), PBE0/cc-pvdz/Amber). X-ray structure PDB ID 1XLW of diethylphosphorylated wild-type BChE Nachon et al. (2005) was used to build the mutants*.

b *Rows in the table are colored to match Figure 3*.

c *Difference in energy between PI and TS2*.

d *Results from Masson and Lushchekina (2016)*.

e *Calculations for steps following formation of PI were not performed due to high-energy barriers of the first step*.

f *Energy barrier for the aging pathway*.

Mutation L286H (mutation #14, in Table 1) resulted in low reactivation. QM/MM calculations show that the energy barrier for this mutant is slightly lower than that for G117H, but the PI is unstable (Table 2). Indeed, the relative positions of H286 and W231 prevent formation of a stable PI: H286 has an unfavorable angle and W231 hinders the shift of the oxy-ethyl group from the diethylphosphate in the plane of the trigonal bipyramid (Figure 3). Replacement of the neighboring, rigid P285 (Figure 2) with a more flexible residue (alanine in our model) improved the orientation of H286 (Table 2). This fits what was observed for self-reactivating bovine BChE (Dafferner et al., 2017), for swine BChE (Brazzolotto et al., 2015), and for mutants generated by the combinatorial approach (Terekhov et al., 2017).

Further improvement in the efficiency of nucleophilic attack could be achieved by introduction of amino acids that facilitate protonation of the histidine during activation of the water molecule and stabilization of the resulting intermediate. In classical catalytic triads, also called “charge-relay systems,” transfer of a proton from histidine to the carboxylate group of glutamic or aspartic acid is unlikely (Steitz and Shulman, 1982; Viragh et al., 2000; Massiah et al., 2001). The role of an acidic residue in such a system is to orientate a histidine, increase its p*K*_a_, and cause charge stabilization of an ion pair between the imidazolium cation and the negatively charged intermediate. A similar arrangement can be introduced for charge stabilization of H286. Geometrically favorable positions for such mutagenesis are positions F329, F357 or V393.

**Figure 5:**
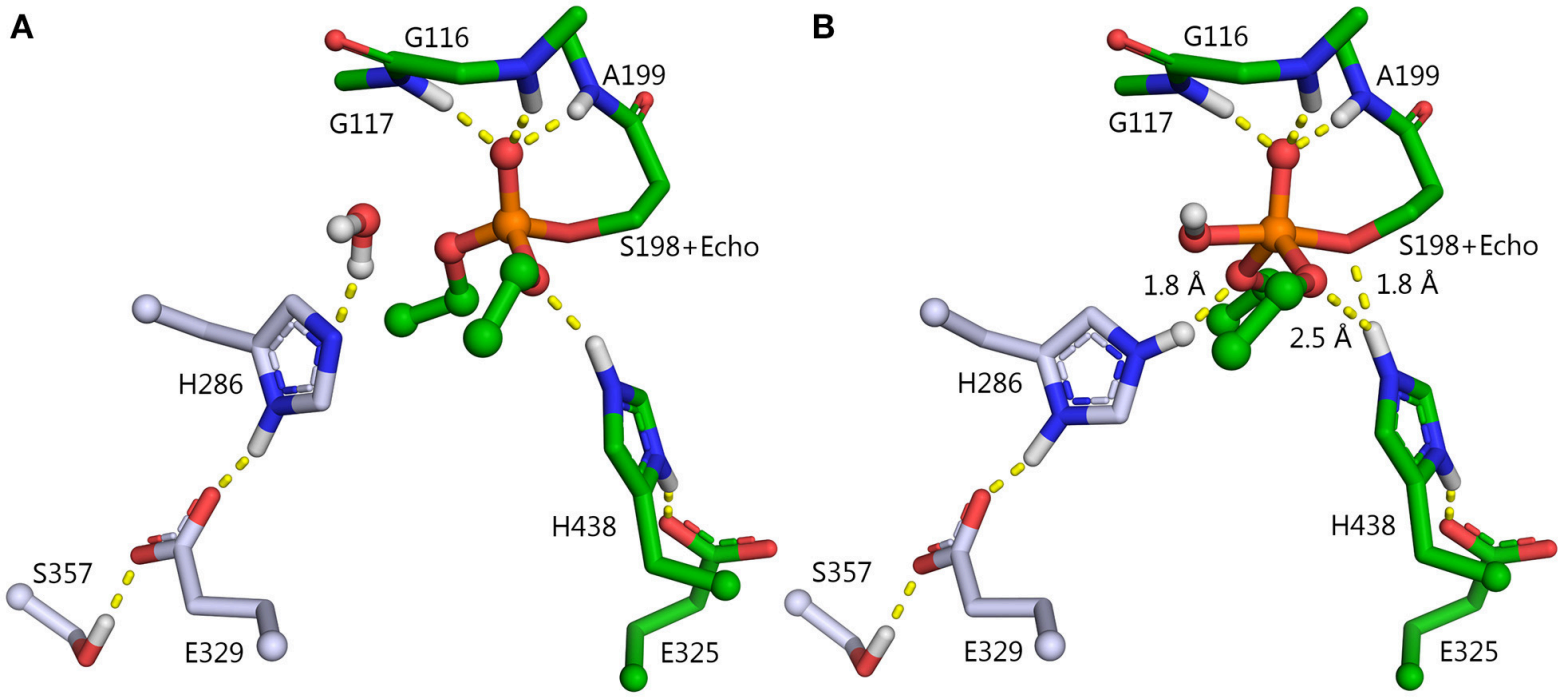
Enzyme-inhibitor conjugate **(A)** and PI **(B)** for the L286H/P285A/F329E/F357S mutant. Carbon atoms of the mutated resides are shown in blue. P285A mutation is not shown.

For our work, we chose F329E and F357S mutations that create a stable hydrogen-bonded network with H286 (Figure 5A). Molecular dynamics simulation of folded enzyme was performed for the quadruple L286H/P285A/F329E/F357S mutant (see Supplementary Information). QM/MM calculations demonstrated a significant decrease in the energy barrier due charge stabilization of the imidazolium cation by E329 and S357 (Table 2 and Figure 4). In the resulting PI, protonated H286 forms a hydrogen bond with the oxygen atom of one of the oxy-ethyl substituents from echothiopate. Unfortunately, this hydrogen bond is favorable for subsequent aging (Figure 5B). Indeed, QM/MM calculation confirmed, that the energy barriers for the reactivation and aging steps are very close (Table 2). Thus, the proposed improvement from L286H could lead to a much faster hydrolysis, but the competing aging process is still a concern.

### Reactivation without competing aging

Figure 3 shows another direction for nucleophilic attack by a water molecule, Direction C. This construct results in an intermediate configuration unfavorable for aging. In this scenario, a pair of mutations, F329H and F398H suppress the hydrophobic pocket, create enough space for a water molecule, and orient the water molecule in a proper position to facilitate hydrolysis of the diethylphospho-adduct.

There is little existing data that bears on this proposal. Double mutant G117H/F329H displays moderate reactivation ability (mutation #9, in Table 1). For position F398 in Table 1, only replacements with isoleucine were considered. This is because most mammalian BChEs have isoleucine in this position (Peng et al., 2015) (see sequence alignment of BChE from 12 species in the Supplemental Information of Peng et al., 2015). This includes self-reactivating bovine BChE (Dafferner et al., 2017).

**Figure 6:**
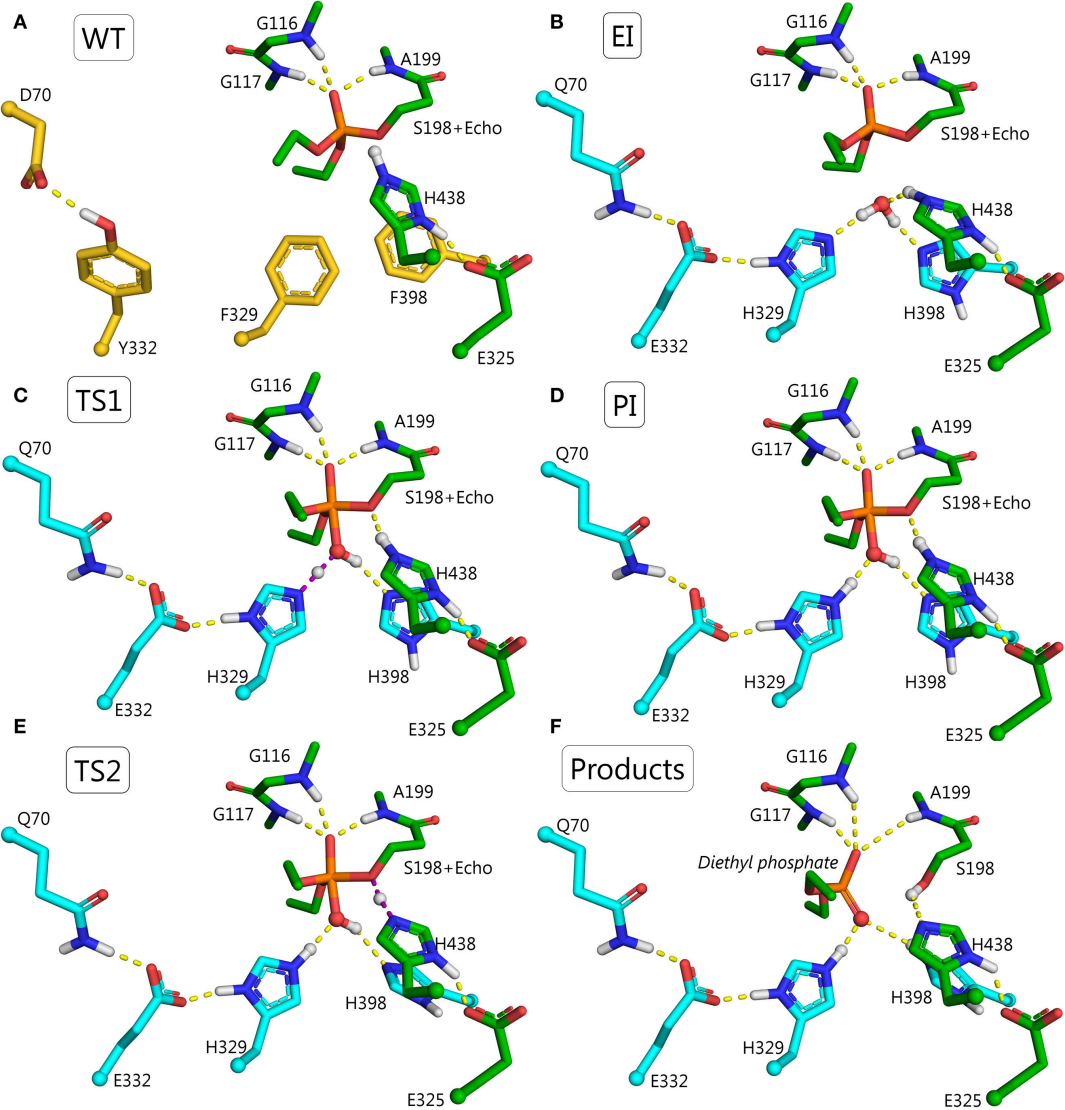
Structures for the proposed F329H/Y332E/D70Q/F398H mutant at various points in the reactivation pathway. **(A)** Wild-type BChE, residues to be mutated are shown in yellow; **(B)** conjugate between echothiopate (Echo) and catalytic serine of F329H/Y332E/D70Q/F398H mutant, mutated residues are colored in cyan; **(C)** first transition state, i.e., nucleophilic attack of the water molecule on the phosphorus atom and transfer of a proton from the water molecule to H329 (violet dashes); **(D)** pentacoordinate intermediate stabilized by hydrogen bonds with H398 and Q70-E332-H329 system; **(E)** second transition state, i.e., transfer of a proton from the catalytic H438 to oxygen of the catalytic S198 (violet dashes) accompanied by transfer of a proton from the hydroxyl group of the PI to H398. **(F)** Products of the reactivation reaction, i.e., diethylphosphate and free mutated BChE with the regenerated operative catalytic triad.

F329H is a more likely candidate for activation of a water molecule than F398H, because F398H is stabilized by the G394 side-chain. The 3D structure of BChE suggests that a charge-stabilizing system for H329 can be introduced by replacing F332 with glutamic acid and D70 with glutamine (Figure 6A). In wild-type BChE, these residues are important both for binding of positively-charged substrates and inhibitors on their way down the active site gorge (Masson et al., 1997b,c), and for stabilization of the Ω-loop that connects the entrance of the gorge to the catalytic binding site (Nicolet et al., 2003). Mutation Y332E/D70Q keeps a carboxylate group and a hydrogen bond at the same locations found in wild-type, hence it should not impair substrate traffic or stability of the protein. This was confirmed by molecular dynamics simulations. In summary, the mutant needed to realize this mechanism of hydrolysis includes 4 amino acid replacements: F329H/Y332E/D70Q/F398H (Figure 6B).

The reactivation pathway for this mutant is presented in Figures 6C–F. The main reasons for lowering the energy barrier are: (1) orientation and activation of the water molecule by H329 and H398; (2) stabilization of the intermediate, TS1, and the imidazolium cation by E332 and Q70; (3) additional stabilization of PI by H398; (4) the configuration of PI is favorable only for proton transfer from the catalytic H438 to the oxygen of the catalytic S198. Dealkylation reactions are unlikely due to long distances (>2.5 Å) between oxygen atoms of both oxy-ethyl groups from diethylphosphate and any proton-donating groups; (5) acceptance of a proton at the last step by H398. The product of the reactivation reaction of echothiophate-inhibited BChE is diethyl phosphoric acid. This product has a p*K*_a_ = 1.39 (Kumler and Eiler, 1943). Modeling of the reactivation reaction for G117H BChE showed that a neighboring water molecule accepted the highly mobile proton from diethylphosphoric acid, forming a hydronium ion (Masson and Lushchekina, 2016). In the current pathway H398 can accept this proton and facilitate formation of reaction products.

These changes in the reactivation pathway allowed the enzyme to reactivate with low energy barriers and made the competing aging reaction unfavorable (see Table 2 and Figure 4). Further developments with this mutant will aim for total abolishment of aging process.

## Conclusion

Computational design of ChE-based bioscavengers is still in its infancy. The keystone for rational conversion of esterase to PTE is to understand the reaction mechanisms of native ChEs and their mutants capable of degrading OPs. As mentioned above, moderate OPase activity can be obtained by increasing the availability of the phosphorus atom of the adduct to a water molecule. This appears to be the mechanism in certain naturally occurring BChEs and in the blowfly carboxylesterase (Jackson et al., 2013; Mabbitt et al., 2016). However, efficient hydrolysis of OP-adducts would seem to require (1) changing the configuration of active center residues and outer shells, (2) creating specific routes for water molecules to attack the phosphorus, and (3) creating specific routes for proton transfer and charge stabilization.

By now, vast amounts of kinetic, crystallographic, and computational data have been accumulated to describe mechanisms of ChE-catalyzed hydrolysis of choline esters, OP-phosphylation, and subsequent reactions, i.e., aging, spontaneous reactivation, and oxime-mediated reactivation. The mechanisms of these reactions are different, depending on the OP structure and enantiomeric configuration of the OP. From these studies it appears that introduction of new groups for the purpose of activating water molecules, directing (orienting) water molecules, stabilizing TS, and stabilizing intermediates will be necessary to change the mechanism of post-phosphylation reactions to favor dephosphylation over dealkylation. Our strategy for the design of novel BChE-based catalytic bioscavengers is to improve dephosphylation enhancing its rate by several orders of magnitude, while suppressing dealkylation.

We have computationally analyzed some BChE mutants, experimentally tested them, and improved their performance by additional mutations. The results suggest that to enhance catalytic efficacy the following issues should be addressed: (a) identification of a geometrically favorable position for a new nucleophile and direction of attack by that nucleophile on the phosphorus of the adduct; (b) introduction of additional mutations for charge stabilization of intermediates and products; and (c) suppression of the competing aging reaction by proper choice of nucleophilic attack direction. These issues indicate that multiple mutations are required. However, multiple mutations may impair folding, stability and catalytic activity of the enzyme. Effects of proposed mutations on the stability and catalytic activity of mutants can be assessed by molecular dynamics simulations and QM/MM calculations, but folding is an issue to be addressed experimentally.

## Author contributions

Most of human BChE mutants in Table 1 were made and expressed by OL. LS and PM performed kinetic studies of these mutants. Quantum mechanics and molecular mechanics supercomputer simulations for analysis of expressed mutants and computer design of new mutations were performed by SL, BG, AN, and SV. The manuscript was written by SL, PM, and LS.

### Conflict of interest statement

The authors declare that the research was conducted in the absence of any commercial or financial relationships that could be construed as a potential conflict of interest.

## Abbreviations

AChE: acetylcholinesterase
BChE: butyrylcholinesterase
ChEs: cholinesterases
CWA: chemical warfare agent
CWNA: chemical warfare nerve agent
EI: enzyme-inhibitor conjugate
MM: molecular modeling
NA: nerve agent
OP: organophosphorus compound
OPase: organophosphate hydrolase
PI: pentacoordinate intermediate
PLL: phosphotriesterase-like lactonase
PON-1: paraoxonase-1
PTE: phosphotriesterase
QM/MM: quantum mechanics/molecular mechanics
TS: transition state
VX: O-ethyl-S-[2-[bis(1-methyl-ethyl)amino]ethyl]methylphosphonothoate.

## References

[B1] Albaret C. Masson P. Broomfield C. A. El Kaim L. Fortier P. L. (1998). Mechanical aspects of the phosphotriesterase activity of human butyrylcholinesterase G117H mutant, in Structure and Function of Cholinesterases and Related Proteins, 1st Edn., eds Doctor B. P. Quinn D. M. Taylot P. Rotundo R. L. (New York, NY: Plenum Press), 399–405.

[B2] Amitay M. Shurki A. (2009). The structure of G117H mutant of butyrylcholinesterase: nerve agents scavenger. Proteins 77, 370–377. 10.1002/prot.22442 19452557

[B3] Amitay M. Shurki A. (2011). Hydrolysis of organophosphate compounds by mutant butyrylcholinesterase: a story of two histidines. Proteins 79, 352–364. 10.1002/prot.22864 21064131

[B4] Ashani Y. Leader H. Aggarwal N. Silman I. Worek F. Sussman J. L.. (2016). *In vitro* evaluation of the catalytic activity of paraoxonases and phosphotriesterases predicts the enzyme circulatory levels required for *in vivo* protection against organophosphate intoxications. Chem. Biol. Interact. 259(Pt B), 252–256. 10.1016/j.cbi.2016.04.039 27163850 PMC5097891

[B5] Bigley A. N. Mabanglo M. F. Harvey S. P. Raushel F. M. (2015). Variants of phosphotriesterase for the enhanced detoxification of the chemical warfare agent VR. Biochemistry 54, 5502–5512. 10.1021/acs.biochem.5b00629 26274608

[B6] Brazzolotto X. Froment M. T. Gessay F. Worek F. Dorandeu F. Nachon F. (2015). Biochemical and structural study of a self-reactivating butyrylcholinesterase after V-type nerve agent inhibition, in 12th International Meeting of Cholinesterases - 6th-Paraoxonase Conference (Elche).

[B7] Brazzolotto X. Igert A. Guillon V. Santoni G. Nachon F. (2017). Bacterial expression of human butyrylcholinesterase as a tool for nerve agent bioscavengers development. Molecules 22:1828. 10.3390/molecules22111828 29077024 PMC6150354

[B8] Broomfield C. A. Lockridge O. Millard C. B. (1999). Protein engineering of a human enzyme that hydrolyzes V and G nerve agents: design, construction and characterization. Chem. Biol. Inter. 119–120, 413–418. 10.1016/s0009-2797(99)00053-8 10421478

[B9] Dafferner A. J. Lushchekina S. Masson P. Xiao G. Schopfer L. M. Lockridge O. (2017). Characterization of butyrylcholinesterase in bovine serum. Chem. Biol. Interact. 266, 17–27. 10.1016/j.cbi.2017.02.004 28189703 PMC5383351

[B10] Doctor B. P. Saxena A. (2005). Bioscavengers for the protection of humans against organophosphate toxicity. Chem. Biol. Interact. 157–158, 167–171. 10.1016/j.cbi.2005.10.024 16293236

[B11] Dorandeu F. Foquin A. Briot R. Delacour C. Denis J. Alonso A.. (2008). An unexpected plasma cholinesterase activity rebound after challenge with a high dose of the nerve agent VX. Toxicology 248, 151–157. 10.1016/j.tox.2008.03.013 18450356

[B12] Eddleston M. Buckley N. A. Eyer P. Dawson A. H. (2008). Management of acute organophosphorus pesticide poisoning. Lancet 371, 597–607. 10.1016/S0140-6736(07)61202-1 17706760 PMC2493390

[B13] Field M. J. Wymore T. W. (2014). Multiscale modeling of nerve agent hydrolysis mechanisms: a tale of two nobel prizes. Phys. Scrip. 89:108004 10.1088/0031-8949/89/10/108004

[B14] Goldenzweig A. Goldsmith M. Hill S. E. Gertman O. Laurino P. Ashani Y.. (2016). Automated structure- and sequence-based design of proteins for high bacterial expression and stability. Mol. Cell 63, 337–346. 10.1016/j.molcel.2016.06.012 27425410 PMC4961223

[B15] Goldsmith M. Aggarwal N. Ashani Y. Jubran H. Greisen P. J. Ovchinnikov S.. (2017). Overcoming an optimization plateau in the directed evolution of highly efficient nerve agent bioscavengers. Protein Eng. Des. Sel. 30, 333–345. 10.1093/protein/gzx003 28159998

[B16] Goldsmith M. Eckstein S. Ashani Y. Greisen P. Jr. Leader H. Sussman J. L.. (2016). Catalytic efficiencies of directly evolved phosphotriesterase variants with structurally different organophosphorus compounds *in vitro*. Arch. Toxicol. 90, 2711–2724. 10.1007/s00204-015-1626-2 26612364

[B17] Hiblot J. Bzdrenga J. Champion C. Chabriere E. Elias M. (2015). Crystal structure of VmoLac, a tentative quorum quenching lactonase from the extremophilic crenarchaeon *Vulcanisaeta moutnovskia*. Sci. Rep. 5:8372. 10.1038/srep08372 25670483 PMC4323659

[B18] Jackson C. J. Liu J.-W. Carr P. D. Younus F. Coppin C. Meirelles T.. (2013). Structure and function of an insect α-carboxylesterase (αEsterase7) associated with insecticide resistance. Proc. Natl. Acad. Sci. U.S.A. 110, 10177–10182. 10.1073/pnas.1304097110 23733941 PMC3690851

[B19] Jacob R. B. Michaels K. C. Anderson C. J. Fay J. M. Dokholyan N. V. (2016). Harnessing nature's diversity: discovering organophosphate bioscavenger characteristics among low molecular weight proteins. Sci. Rep. 6:37175. 10.1038/srep37175 27845442 PMC5109037

[B20] Jacquet P. Daudé D. Bzdrenga J. Masson P. Elias M. Chabrière E. (2016). Current and emerging strategies for organophosphate decontamination: special focus on hyperstable enzymes. Environ. Sci. Pollut. Res. 23, 8200–8218. 10.1007/s11356-016-6143-1 26832878

[B21] Jarv J. (1984). Stereochemical aspects of cholinesterase catalysis. Bioorg. Chem. 12, 259–278. 10.1016/0045-2068(84)90010-5

[B22] Kovarik Z. Macek Hrvat N. Katalinic M. Sit R. K. Paradyse A. Zunec S.. (2015). Catalytic soman scavenging by the Y337A/F338A acetylcholinesterase mutant assisted with novel site-directed aldoximes. Chem. Res. Toxicol. 28, 1036–1044. 10.1021/acs.chemrestox.5b00060 25835984 PMC4791098

[B23] Kulakova A. Lushchekina S. Grigorenko B. Nemukhin A. (2015). Modeling reactivation of the phosphorylated human butyrylcholinesterase by QM(DFTB)/MM calculations. J. Theor. Comp. Chem. 14:1550051 10.1142/s0219633615500510

[B24] Kumler W. D. Eiler J. J. (1943). The acid strength of mono and diesters of phosphoric acid. The n-alkyl esters from methyl to butyl, the esters of biological importance, and the natural guanidine phosphoric acids. J. Am. Chem. Soc. 65, 2355–2361. 10.1021/ja01252a028

[B25] Lenz D. E. Maxwell D. M. Koplovitz I. Clark C. R. Capacio B. R. Cerasoli D. M.. (2005). Protection against soman or VX poisoning by human butyrylcholinesterase in guinea pigs and cynomolgus monkeys. Chem. Biol. Interact. 157–158, 205–210. 10.1016/j.cbi.2005.10.025 16289064

[B26] Lockridge O. (2015). Review of human butyrylcholinesterase structure, function, genetic variants, history of use in the clinic, and potential therapeutic uses. Pharmacol. Ther. 148, 34–46. 10.1016/j.pharmthera.2014.11.011 25448037

[B27] Lockridge O. Blong R. M. Masson P. Froment M. T. Millard C. B. Broomfield C. A. (1997). A single amino acid substitution, Gly117His, confers phosphotriesterase (organophosphorus acid anhydride hydrolase) activity on human butyrylcholinesterase. Biochemistry 36, 786–795. 10.1021/bi961412g 9020776

[B28] Mabbitt P. D. Correy G. J. Meirelles T. Fraser N. J. Coote M. L. Jackson C. J. (2016). Conformational disorganization within the active site of a recently evolved organophosphate hydrolase limits its catalytic efficiency. Biochemistry 55, 1408–1417. 10.1021/acs.biochem.5b01322 26881849

[B29] Massiah M. A. Viragh C. Reddy P. M. Kovach I. M. Johnson J. Rosenberry T. L.. (2001). Short, strong hydrogen bonds at the active site of human acetylcholinesterase: proton NMR studies. Biochemistry 40, 5682–5690. 10.1021/bi010243j 11341833

[B30] Masson P. (2016a). Nerve agents: catalytic scavengers, alternative approach for medical countermeasures, in Chemical Warfare Toxicology, eds Worek F. Jenner J. Thiermann H. (Cambridge, UK: Royal Society of Chemistry Pub), 43–81.

[B31] Masson P. (2016b). Novel approaches in prophylaxis/pretreatment and treatment of organophosphorus poisoning. Phosph. Sulfur Silicon Relat. Elements 191, 1433–1443. 10.1080/10426507.2016.1211652

[B32] Masson P. Fortier P.-L. Albaret C. Froment M.-T. Bartels F. C. Lockridge O. (1997a). Aging of di-isopropyl-phosphorylated human butyrylcholinesterase. Biochem. J. 327, 601–607. 10.1042/bj3270601 9359435 PMC1218835

[B33] Masson P. Froment M. T. Bartels C. F. Lockridge O. (1997b). Importance of aspartate-70 in organophosphate inhibition, oxime re-activation and aging of human butyrylcholinesterase. Biochem. J. 325 (Pt 1), 53–61. 9224629 10.1042/bj3250053 PMC1218528

[B34] Masson P. Legrand P. Bartels C. F. Froment M.-T. Schopfer L. M. Lockridge O. (1997c). Role of aspartate 70 and tryptophan 82 in binding of succinyldithiocholine to human butyrylcholinesterase. Biochemistry 36, 2266–2277. 10.1021/bi962484a 9047329

[B35] Masson P. Lushchekina S. V. (2016). Emergence of catalytic bioscavengers against organophosphorus agents. Chem. Biol. Interact. 259(Pt B), 319–326. 10.1016/j.cbi.2016.02.010 26899146

[B36] Masson P. Nachon F. (2017). Cholinesterase reactivators and bioscavengers for pre- and post-exposure treatments of organophosphorus poisoning. J. Neurochem. 142(Suppl. 2), 26–40. 10.1111/jnc.14026 28542985

[B37] Masson P. Nachon F. Broomfield C. A. Lenz D. E. Verdier L. Schopfer L. M.. (2008). A collaborative endeavor to design cholinesterase-based catalytic scavengers against toxic organophosphorus esters. Chem. Biol. Interact. 175, 273–280. 10.1016/j.cbi.2008.04.005 18508040

[B38] Masson P. Nachon F. Lockridge O. (2010). Structural approach to the aging of phosphylated cholinesterases. Chem. Biol. Interact. 187, 157–162. 10.1016/j.cbi.2010.03.027 20338153

[B39] Millard C. B. Lockridge O. Broomfield C. A. (1995). Design and expression of organophosphorus acid anhydride hydrolase activity in human butyrylcholinesterase. Biochemistry 34, 15925–15933. 10.1021/bi00049a007 8519749

[B40] Millard C. B. Lockridge O. Broomfield C. A. (1998). Organophosphorus acid anhydride hydrolase activity in human butyrylcholinesterase: synergy results in a somanase. Biochemistry 37, 237–247. 10.1021/bi972057c 9425044

[B41] Mumford H. Docx C. J. Price M. E. Green A. C. Tattersall J. E. H. Armstrong S. J. (2013). Human plasma-derived BuChE as a stoichiometric bioscavenger for treatment of nerve agent poisoning. Chem. Biol. Interact. 203, 160–166. 10.1016/j.cbi.2012.08.018 22981459

[B42] Myhrer T. Aas P. (2016). Pretreatment and prophylaxis against nerve agent poisoning: are undesirable behavioral side effects unavoidable? Neurosci. Biobehav. Rev. 71, 657–670. 10.1016/j.neubiorev.2016.10.017 27773692

[B43] Nachon F. Asojo O. A. Borgstahl G. E. Masson P. Lockridge O. (2005). Role of water in aging of human butyrylcholinesterase inhibited by echothiophate: the crystal structure suggests two alternative mechanisms of aging. Biochemistry 44, 1154–1162. 10.1021/bi048238d 15667209

[B44] Nachon F. Brazzolotto X. Trovaslet M. Masson P. (2013). Progress in the development of enzyme-based nerve agent bioscavengers. Chem. Biol. Interact. 206, 536–544. 10.1016/j.cbi.2013.06.012 23811386

[B45] Nachon F. Carletti E. Wandhammer M. Nicolet Y. Schopfer L. M. Masson P.. (2011). X-ray crystallographic snapshots of reaction intermediates in the G117H mutant of human butyrylcholinesterase, a nerve agent target engineered into a catalytic bioscavenger. Biochem. J. 434, 73–82. 10.1042/bj20101648 21091433

[B46] Nemukhin A. V. Kulakova A. M. Lushchekina S. V. Ermilov A. Y. Varfolomeev S. D. (2015). Modeling chemical transformations at the active sites of cholinesterases by quantum-based simulations. Mosc. Univ. Chem. Bull. 70, 274–277. 10.3103/S0027131415060061

[B47] Nicolet Y. Lockridge O. Masson P. Fontecilla-Camps J. C. Nachon F. (2003). Crystal structure of human butyrylcholinesterase and of its complexes with substrate and products. J. Biol. Chem. 278, 41141–41147. 10.1074/jbc.M210241200 12869558

[B48] Onder S. David E. Tacal O. Schopfer L. M. Lockridge O. (2017). Hupresin retains binding capacity for butyrylcholinesterase and acetylcholinesterase after sanitation with sodium hydroxide. Front. Pharmacol. 8:713. 10.3389/fphar.2017.00713 29066970 PMC5641355

[B49] Pashirova T. N. Zueva I. V. Petrov K. A. Babaev V. M. Lukashenko S. S. Rizvanov I. K.. (2017). Nanoparticle-delivered 2-PAM for rat brain protection against paraoxon central toxicity. ACS Appl. Mater. Interfaces 9, 16922–16932. 10.1021/acsami.7b04163 28504886

[B50] Patocka J. (2017). What killed Kim Jong-nam? Was it the agent VX. Mil. Med. Sci. Lett. 86, 1–4.

[B51] Peng H. Brimijoin S. Hrabovska A. Targosova K. Krejci E. Blake T. A.. (2015). Comparison of 5 monoclonal antibodies for immunopurification of human butyrylcholinesterase on Dynabeads: KD values, binding pairs, and amino acid sequences. Chem. Biol. Interact. 240, 336–345. 10.1016/j.cbi.2015.08.024 26343001 PMC5095618

[B52] Poyot T. Nachon F. Froment M. T. Loiodice M. Wieseler S. Schopfer L. M.. (2006). Mutant of *Bungarus fasciatus* acetylcholinesterase with low affinity and low hydrolase activity toward organophosphorus esters. Biochim. Biophys. Acta 1764, 1470–1478. 10.1016/j.bbapap.2006.07.008 16962835

[B53] Radić Z. Dale T. Kovarik Z. Berend S. Garcia E. Zhang L.. (2013). Catalytic detoxification of nerve agent and pesticide organophosphates by butyrylcholinesterase assisted with non-pyridinium oximes. Biochem. J. 450, 231–242. 10.1042/bj20121612 23216060 PMC4772673

[B54] Restaino O. F. Borzacchiello M. G. Scognamiglio I. Porzio E. Manco G. Fedele L.. (2017). Boosted large-scale production and purification of a thermostable archaeal phosphotriesterase-like lactonase for organophosphate decontamination. J. Ind. Microbiol. Biotechnol. 44, 363–375. 10.1007/s10295-016-1892-x 28074318

[B55] Rice H. Mann T. M. Armstrong S. J. Price M. E. Green A. C. Tattersall J. E. (2016). The potential role of bioscavenger in the medical management of nerve-agent poisoned casualties. Chem. Biol. Interact. 259(Pt B), 175–181. 10.1016/j.cbi.2016.04.038 27144491

[B56] Schopfer L. M. Boeck A. T. Broomfield C. A. Lockridge O. (2004). Mutants of human butyrylcholinesterase with organophosphate hydrolase activity; evidence that His117 is a general base catalyst for hydrolysis of echothiophate. J. Med. Chem. Biol. Radiol. Def. 2, 1–21.

[B57] Steitz T. A. Shulman R. G. (1982). Crystallographic and NMR studies of the serine proteases. Annu. Rev. Biophys. Bioeng. 11, 419–444. 10.1146/annurev.bb.11.060182.002223 7049067

[B58] Terekhov S. S. Smirnov I. V. Stepanova A. V. Bobik T. V. Mokrushina Y. A. Ponomarenko N. A.. (2017). Microfluidic droplet platform for ultrahigh-throughput single-cell screening of biodiversity. Proc. Natl. Acad. Sci. U.S.A. 114, 2550–2555. 10.1073/pnas.1621226114 28202731 PMC5347554

[B59] Valiev M. Bylaska E. J. Govind N. Kowalski K. Straatsma T. P. Van Dam H. J. J. (2010). NWChem: a comprehensive and scalable open-source solution for large scale molecular simulations. Comput. Phys. Commun. 181, 1477–1489. 10.1016/j.cpc.2010.04.018

[B60] Viragh C. Harris T. K. Reddy P. M. Massiah M. A. Mildvan A. S. Kovach I. M. (2000). NMR evidence for a short, strong hydrogen bond at the active site of a cholinesterase. Biochemistry 39, 16200–16205. 10.1021/bi0022644 11123949

[B61] Voevodin V. V. Zhumatiy S. A. Sobolev S. I. Antonov A. S. Bryzgalov P. A. Nikitenko D. A. (2012). Practice of “Lomonosov” supercomputer. Open Syst. J. 7, 36–39.

[B62] Wandhammer M. Carletti E. van der Schans M. Gillon E. Nicolet Y. Masson P.. (2011). Structural study of the complex stereoselectivity of human butyrylcholinesterase for the neurotoxic V-agents. J. Biol. Chem. 286, 16783–16789. 10.1074/jbc.M110.209569 21454498 PMC3089521

[B63] Wille T. Neumaier K. Koller M. Ehinger C. Aggarwal N. Ashani Y.. (2016). Single treatment of VX poisoned guinea pigs with the phosphotriesterase mutant C23AL: intraosseous versus intravenous injection. Toxicol. Lett. 258, 198–206. 10.1016/j.toxlet.2016.07.004 27397758

[B64] Worek F. Thiermann H. Wille T. (2016a). Catalytic bioscavengers in nerve agent poisoning: a promising approach? Toxicol. Lett. 244, 143–148. 10.1016/j.toxlet.2015.07.012 26200600

[B65] Worek F. Wille T. Koller M. Thiermann H. (2016b). Toxicology of organophosphorus compounds in view of an increasing terrorist threat. Arch. Toxicol. 90, 2131–2145. 10.1007/s00204-016-1772-1 27349770

[B66] Yao Y. Liu J. Zhan C.-G. (2012). Why does the G117H mutation considerably improve the activity of human butyrylcholinesterase against sarin? Insights from quantum mechanical/molecular mechanical free energy calculations. Biochemistry 51, 8980–8992. 10.1021/bi3009246 23092211 PMC3499032

