# Supplementary material for: Optimization of Cholinesterase-Based Catalytic Bioscavengers Against Organophosphorus Agents
Source: Front Pharmacol. 2018 Mar 13;9:211. doi: 10.3389/fphar.2018.00211 (PMC5859046; doi:10.3389/fphar.2018.00211)
Supplement: Supplementary file 1 [file DataSheet1.PDF]

Molecular modeling methods

Butyrylcholinesterase mutants were built from X-ray structure PDB ID 1XLW of diethylphosphorylated wild-type BChE (Nachon et al., 2005) solved at 2.10 Å resolution. Missing amino acids D378 and D379 were taken from another X-ray structure of BChE, PDB ID: 2PM8 (Ngamelue et al., 2007). Hydrogen atoms were added with respect of hydrogen bonding network by Reduce software (Word et al., 1999). For each mutant side chains were changed manually, and two systems were prepared: an *apo* state and with diethylphosphorylated serine and protonated H438 (See Figure 2, main text). Water molecules recognized in the crystal structure were included to the model system, and TIP3P water molecules were added, forming a box with boundaries exceeding 10 Å from the protein by means of VMD *solvate* module. Sodium and chloride ions were added up to 0.15 M ion concentration using VMD *autoionize* module. CHARMM36 force field (Best et al., 2012) was used, diethylphosphorylated serine residue was parameterized with CGenFF service (Vanommeslaeghe and MacKerell, 2012; Vanommeslaeghe et al., 2012).

Molecular dynamics simulations were performed with the NAMD 2.11 program (Phillips et al., 2005) at the Lomonosov Moscow State University supercomputer (Sadovnichy et al., 2013). During MD simulations, systems were maintained at constant temperature 298 K and under pressure 1 atm (NPT ensemble) by using Langevin dynamics and Nosé-Hoover barostat. Periodical boundary conditions and PME electrostatics were applied. Prior to QM/MM calculations and productive MD runs, optimization of mutated residues and solvent equilibration were performed during 2000 steps of minimization and 1 ns equilibrating run was performed with the protein coordinates fixed (except for the mutated and two neighboring residues), and fully minimized during 2000 steps. For QM/MM calculations protein with solvation shell of 1653 water molecules was cut from the minimized system.

QM/MM calculations were performed with NwChem 6.5 software (Valiev et al., 2010), the hydrogen link atom scheme to treat the QM-MM boundary, and the electronic embedding QM/MM strategy were applied. QM calculations were carried out with the density functional theory version PBE0/cc-pvdz. The MM subsystem was modeled with the AMBER force field (Case et al., 2005). Quantum subsystem included all major amino acids of the active site: diethylphosphorylated S198, H438, E325 — the catalytic triad, G116, G117, A199 — the oxyanion hole, other important residues side chains, Glu197 and Ser224, reacting water molecule and mutated residues (except for A285, which doesn’t participate in the reaction). Quantum subsystems for different mutants are presented in Figure SI1. Total size of the systems was ~13300 atoms.

Structures of the enzyme-inhibitor complexes (EI), reaction products (Prod) and pentacoordinate intermediates (PI) were obtained in series of unconstrained QM/MM minimizations following scans along the appropriate reaction coordinates (see Figure 3, main text).

L286H

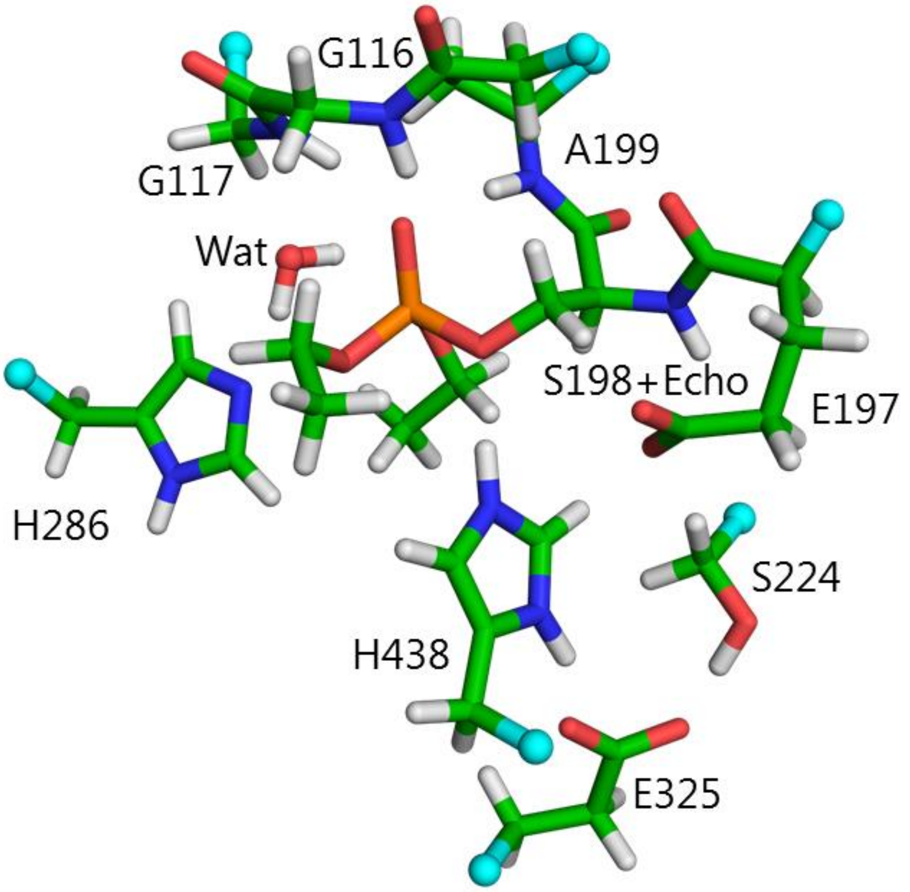

L286H/P285A/F329E/F357S

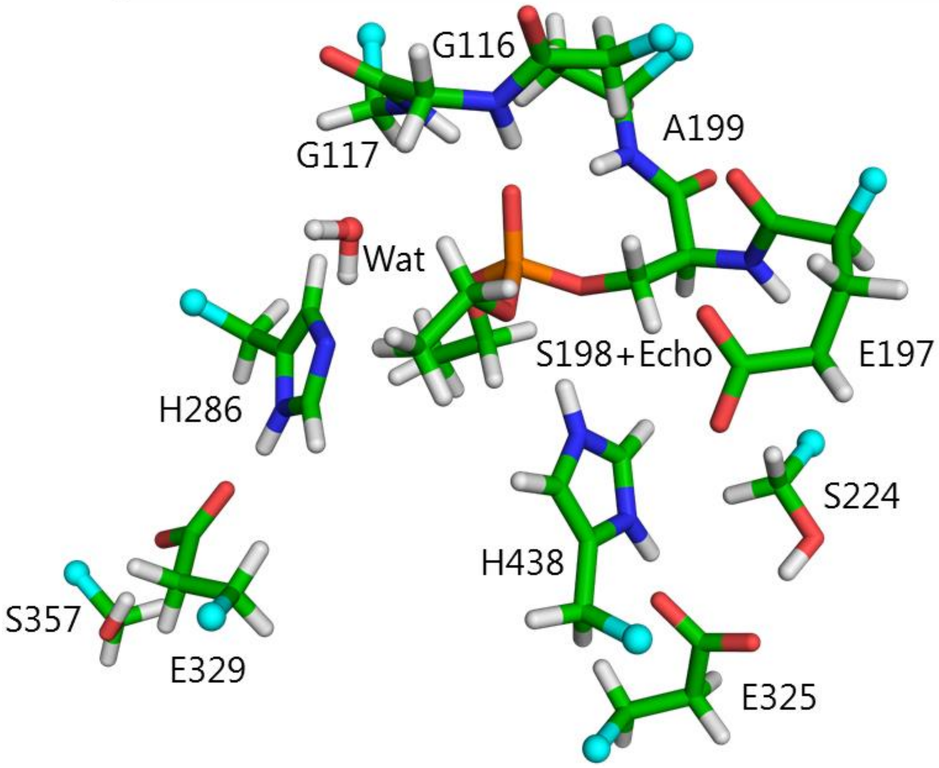

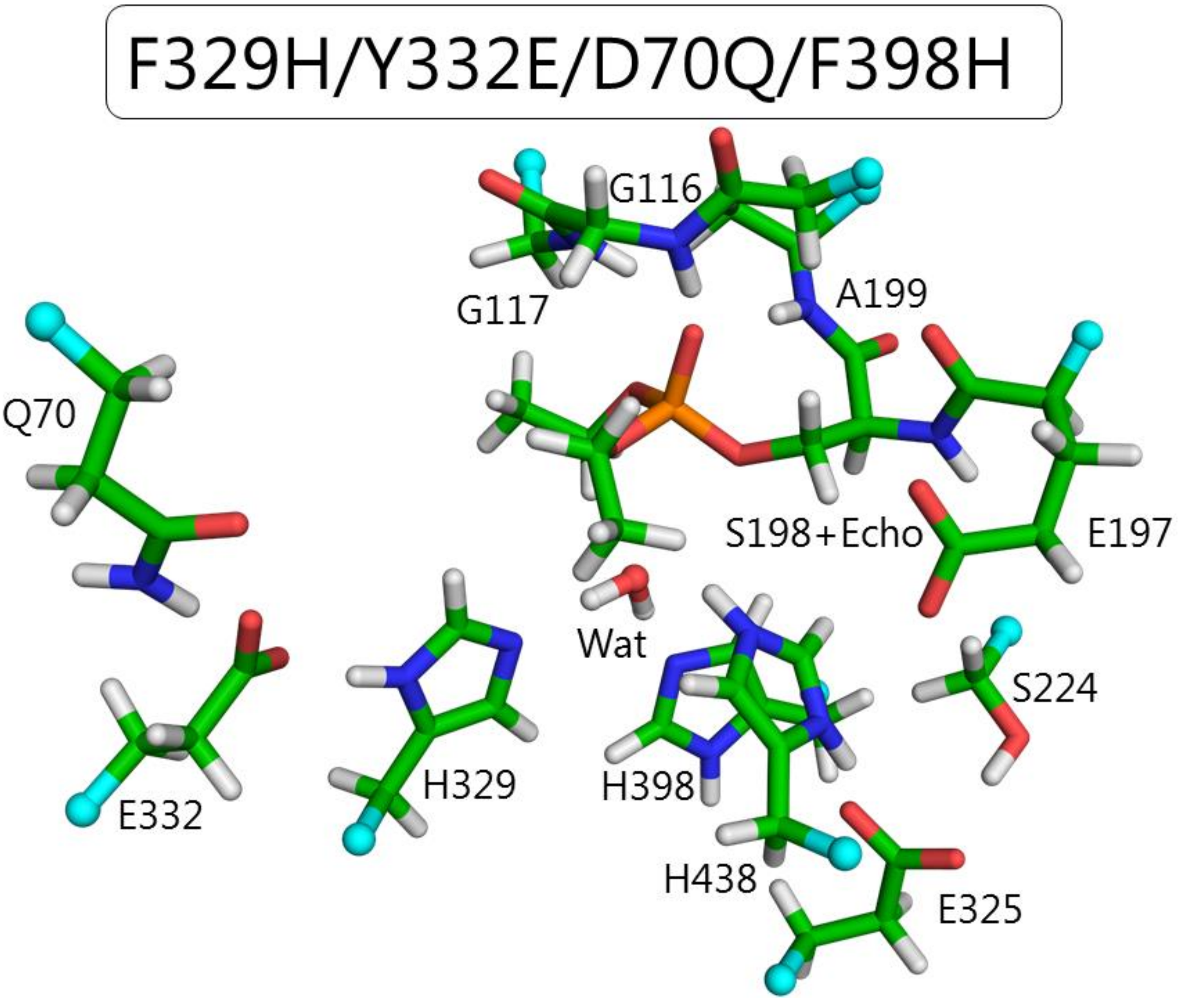

Figure SI1. Quantum subsystems for QM/MM calculations for mutants L286H (106 atoms), L286H/P285A/F329E/F357S (120 atoms), F329H/Y332E/D70Q/F398H (138 atoms). Link atoms are shown cyan.

For the equilibrated systems, productive 100 ns MD runs were performed, distances between catalytic triad residues side chains (Ser198, His438 and Glu325) were controlled. Figure SI2 shows distances over MD trajectory for L286H/P285A/F329E/F357S mutant, distances in catalytic triad show good stability of the system.

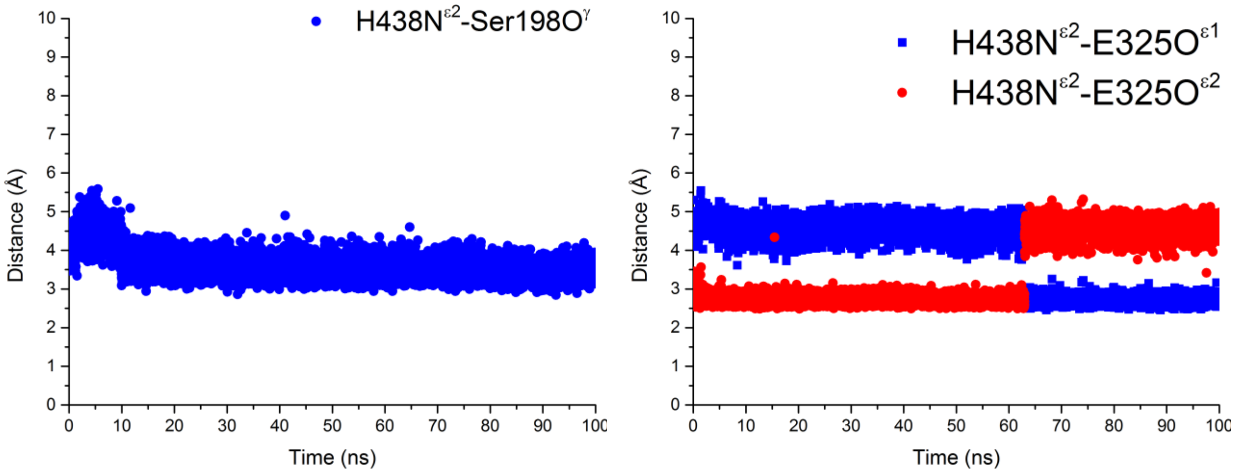

Figure SI2. Principle distances in the L286H/P285A/F329E/F357S mutant catalytic triad over MD trajectory.

References

Best, R.B., Zhu, X., Shim, J., Lopes, P.E.M., Mittal, J., Feig, M., et al. (2012). Optimization of the additive CHARMM all-atom protein force field targeting improved sampling of the backbone ϕ, ψ and side-chain $\chi 1$ and $\chi 2$ dihedral angles. *Journal of Chemical Theory and Computation* 8(9)**,** 3257-3273. doi: 10.1021/ct300400x.

Case, D.A., Cheatham, T.E., 3rd, Darden, T., Gohlke, H., Luo, R., Merz, K.M., Jr., et al. (2005). The Amber biomolecular simulation programs. *J Comput Chem* 26(16)**,** 1668-1688. doi: 10.1002/jcc.20290.

Nachon, F., Asojo, O.A., Borgstahl, G.E., Masson, P., and Lockridge, O. (2005). Role of water in aging of human butyrylcholinesterase inhibited by echothiophate: the crystal structure suggests two alternative mechanisms of aging. *Biochemistry* 44(4)**,** 1154-1162. doi: 10.1021/bi048238d.

Ngamelue, M.N., Homma, K., Lockridge, O., and Asojo, O.A. (2007). Crystallization and X-ray structure of full-length recombinant human butyrylcholinesterase. *Acta Crystallogr Sect F Struct Biol Cryst Commun* 63(Pt 9)**,** 723-727. doi: 10.1107/S1744309107037335.

Phillips, J.C., Braun, R., Wang, W., Gumbart, J., Tajkhorshid, E., Villa, E., et al. (2005). Scalable molecular dynamics with NAMD. *Journal of Computational Chemistry* 26(16)**,** 1781-1802. doi: 10.1002/jcc.20289.

Sadovnichy, V., Tikhonravov, A., Voevodin, V., and Opanasenko, V. (2013). ""Lomonosov": supercomputing at Moscow State University," in *Contemporary High Performance Computing: From Petascale toward Exascale,* ed. J.S. Vetter. (Boca Raton, USA: CRC Press), 283-307.

Valiev, M., Bylaska, E.J., Govind, N., Kowalski, K., Straatsma, T.P., Van Dam, H.J.J., et al. (2010). NWChem: A comprehensive and scalable open-source solution for large scale molecular simulations. *Computer Physics Communications* 181(9)**,** 1477-1489. doi: 10.1016/j.cpc.2010.04.018.

Vanommeslaeghe, K., and MacKerell, A.D., Jr. (2012). Automation of the CHARMM General Force Field (CGenFF) I: bond perception and atom typing. *J Chem Inf Model* 52(12)**,** 3144-3154. doi: 10.1021/ci300363c.

Vanommeslaeghe, K., Raman, E.P., and MacKerell, A.D., Jr. (2012). Automation of the CHARMM General Force Field (CGenFF) II: assignment of bonded parameters and partial atomic charges. *J Chem Inf Model* 52(12)**,** 3155-3168. doi: 10.1021/ci3003649.

Word, J.M., Lovell, S.C., Richardson, J.S., and Richardson, D.C. (1999). Asparagine and glutamine: using hydrogen atom contacts in the choice of side-chain amide orientation. *Journal of Molecular Biology* 285(4)**,** 1735-1747. doi: 10.1006/jmbi.1998.2401.
